# GAS5 Long Noncoding RNA Regulates CD20 Expression and Rituximab Response

**DOI:** 10.34172/apb.025.45822

**Published:** 2025-10-11

**Authors:** Mahbubeh Rojhannezhad, Zahra Abedi Kichi, Abbas Nikravesh, Mehrdad Behmanesh

**Affiliations:** ^1^Department of Genetics, Faculty of Biological Sciences, Tarbiat Modares University, Tehran, Iran; ^2^Institute for Cardiovascular Prevention (IPEK), Ludwig-Maximilians University Munich, Munich, Germany; ^3^Department of Medical Biotechnology & Molecular Sciences, Faculty of Medicine, North Khorasan University of Medical Sciences, Bojnurd, Iran

**Keywords:** Apoptosis, B-cell lymphoma, CD20, GAS5, Rituximab response

## Abstract

**Purpose:**

Rituximab is the primary treatment for non-Hodgkin lymphoma (NHL), one of the most common cancers globally. One of the main challenges associated with rituximab therapy is the decline in its effectiveness over time. Several suggested potential reasons for this therapeutic resistance exist, including the downregulation of CD20 expression. Recently, the focus has shifted to long non-coding RNAs (lncRNAs) like growth arrest specific 5 (GAS5) for their involvement in various physiological functions and their potential role in the response rate to anticancer drugs. In this study, we aimed to investigate the regulatory effect of GAS5 on CD20 expression and the response of cancer cells to rituximab.

**Methods:**

Using the Raji cell model, we assessed the impact of GAS5 knockdown on CD20 expression and the response to rituximab through RT-qPCR assay. Western blot analysis, caspase-3 activity, and ROS assay were conducted to evaluate protein expression levels, apoptosis, and oxidative stress, respectively.

**Results:**

In silico analysis predicted interactions between GAS5 and regulatory proteins associated with CD20. GAS5 knockdown increased CD20 and STAT3 expression while decreasing SMAD2 levels and apoptosis. It also reduced generation of reactive oxygen species (ROS) and enhanced autophagy. However, combining GAS5 knockdown with rituximab elevated apoptosis and autophagy while further reducing ROS. These findings suggest an indirect regulatory role for GAS5 in CD20 expression, potentially via modulation of CD20-associated regulatory proteins. Nonetheless, the study has limitations, including reliance on a single cell line and the assessment of direct apoptosis only.

**Conclusion:**

These findings highlight a complex interplay between GAS5, CD20, rituximab, and cellular pathways, underscoring the significance of understanding these interactions to enhance cancer therapy outcomes.

## Introduction

 Cancerremains a challenging opponent in medical research due to its complex molecular makeup. Non-Hodgkin’s lymphoma (NHL), a heterogeneous malignancy, is considered the seventh most common cancer, which originates from B-cells, T-cells, and natural killer (NK) cells.^1^ The therapeutic landscape against cancers such as NHL was marked by the advent of rituximab, a chimeric monoclonal antibody targeting the CD20 molecule. Rituximab’s impact is particularly evident in the context of NHL, and its addition to chemotherapy regimens has significantly improved patient survival, especially in cases of diffuse large B-cell lymphoma. However, not all patients respond favorably to this therapy, with approximately 30% failing to achieve the desired outcome.^2^ Rituximab targets B cells by multiple pathways, including antibody-dependent cell-mediated cytotoxicity (ADCC), complement-dependent cytotoxicity (CDC), and direct mechanisms that involve intracellular signaling effects through CD20 binding.^3^ CD20, a distinguishing marker of B cells, is involved in the regulation of cell proliferation, differentiation, and initiating intracellular signals. A significant concern in the context of rituximab therapy is the observation that its efficacy diminishes throughout treatment. Several potential mechanisms for this therapeutic resistance have been identified, including the down-regulation of CD20 expression and the stimulation of complement-modulating proteins on the surface of malignant cells.^4^ Understanding these resistance mechanisms is pivotal to improving the overall efficacy of rituximab-based therapies, such as identifying methods to enhance CD20 expression via its regulatory proteins, which could be beneficial.

 In the quest to decode cancer mysteries, our focus has shifted to a particular category of molecules, long non-coding RNAs (lncRNAs), implicated in the regulation of cancer initiation, development, and progression, as well as drug response and resistance due to their ability to regulate various cellular paradigms epigenetically. Multiple studies over the past several years have discussed the importance of lncRNAs in the pathophysiology of all subtypes of lymphoma.^1,5,6^ These adaptable molecules function as oncogenes or tumor suppressors in several cancers, including B-cell NHL subtypes. Their importance in B-cell malignancies, which includes both normal B-cell development and the pathogenesis of B-cell cancers, is becoming clear.^1,7^ Growth arrest specific 5 (GAS5) has emerged as a notable participant in the pantheon of lncRNAs. GAS5, which is distinguished by its length and complicated transcriptional control, has been implicated in various physiological functions. While it is known to be upregulated during growth arrest induced by serum deprivation, its expression takes on a different narrative in numerous cancer types, where it is down-regulated in most cancers, including diffuse large B-cell lymphoma (DLBCL), underlining its potential role as a tumor suppressor. GAS5 operates through different fundamental modes of action, which involve direct connection to target genes primarily at the post-transcriptional level and indirect binding to regulatory proteins, resulting in subsequent modulation of gene transcription. GAS5 is involved in a wide range of cellular processes, including survival, apoptosis, cell cycle regulation, autophagy, immune responses, and drug sensitivity.^8,9^ Its dysregulation has been linked to the pathophysiology of B-cell malignancies, especially DLBCL. Notably, GAS5 has shown diagnostic potential in distinguishing DLBCL cases.^10^ To build on this, our in silico analysis suggests that GAS5 may interact with regulatory proteins associated with CD20, adding another layer of relevance to its role in rituximab response and B-cell lymphoma biology.

 The current study provides valuable insights into the fundamental aspects of cancer research. We utilized the Raji cell model, a CD20-positive human Burkitt lymphoma cell line, to investigate the response of Raji cells to rituximab after down-regulating GAS5. We also assessed the impact of GAS5 downregulation on CD20 expression. We aim to contribute to the expanding knowledge concerning the intricate interactions among lncRNAs, particularly GAS5, CD20, and rituximab.

## Materials and Methods

###  In silico analysis

 Several online web servers, including Lncrna2target v3, LncRRIsearch v1, Harmonizome 3, and hTFtarget, were employed to explore the potential interaction between GAS5 and CD20, as well as CD20 transcription factors.^11-16^

###  Design of GAS5-specific DNAzyme

 DNAzymes, single-stranded DNA molecules approximately 35 nucleotides in length, serve as catalytic tools designed to downregulate the expression of specific target genes. Oligo 7 software (DBA Oligo, USA), and NCBI-BLAST were used to design two GAS5-specific oligonucleotides based on the homology searched sequence. Secondary structure formation within the target RNA regions was evaluated using the RNAfold web server.^17^ The Scramble DNAzymes were also designed and used in the control group of the experiments. The sequence of DNAzymes is listed in Table S1 (Supplementary file 1).

###  Cell culture and treatment

 Human Raji B cells were purchased from the Pasteur Institute (Iran). This cell line was cultured in a Roswell Park Memorial Institute 1640 medium (RPMI 1640, Gibco, Thermo Fisher Scientific, USA) supplemented with 2 mM L-glutamine, 1% penicillin/streptomycin, and 10% fetal bovine serum (Gibco), and incubated at 37 °C in a humidified atmosphere containing 5% CO2. Raji cells were exposed to rituximab (Aryogen Co. Iran) with doses of 5, 10, 20, and 40 µg /mL for 6, 12, 24, and 48h to obtain the best dose and time point for downstream analyses by the MTT method using a plate reader (BioTek Instruments ELX800, USA).

###  RNA extraction and qPCR

 Total RNA was extracted using RiboEX^TM^ solution (GeneAll, Korea), according to the manufacturer’s instructions. Three µg of RNA was reverse-transcribed with a cDNA reverse transcription kit according to the manufacturer’s instructions (BioFACT^TM^ RT-Kit, Korea). Gene expression levels were quantified using the quantitative PCR (qPCR) technique by a StepOne^TM^ PCR system (Applied Biosystem/MDS SCIEX, USA), using the qPCR master mix (EvaGreen® qPCR master mix, Solis Bio Dyne, Estonia). Relative gene expression analysis was conducted using the Livak method (2^−ΔCt^), with β-actin serving as the internal control.^18^ All experiments were performed in three biological replicates. Primer sequences are reported in Table S2.

###  Cell transfection

 Raji cells were transfected with two designed DNAzymes simultaneously (5 nM) for effective knockdown of GAS5 lncRNA, or with a scrambled DNAzyme, by Lipofectamine 2000 transfection reagent (Invitrogen). After four hours, cells were treated with rituximab (10 µg/mL). RNA extraction and subsequent cDNA synthesis were carried out as previously described, around 28 hours after transfection. The efficiency of GAS5 knockdown was validated by qPCR.

###  Flow cytometry assay

 The apoptosis analysis was conducted using a phosphatidylserine detection kit, which includes Annexin V-fluorescein isothiocyanate (Annexin V-FITC) combined with propidium iodide (PI) to assess plasma membrane integrity. In this experiment, Raji cells were cultured, treated with rituximab, and transfected with GAS5 DNAzyme as previously described. The treated and untreated cells (1 × 10^6^ cells/mL) were harvested, washed with PBS, and then suspended in 100 μL of binding buffer containing FITC-Annexin V and PI solution following the manufacturer’s instructions (BD Biosciences, San Jose, CA, USA). The apoptotic cell population was analyzed using a BD FACSCalibur Flow cytometer and quantified using Flow Jo software (Ashland, OR, USA).

###  Western blot analysis

 Proteins were isolated from Raji cells by employing RIPA lysis buffer, consisting of 50 mM Tris, pH 7.4, 150 mM NaCl, 1% Igepal, 0.5% sodium deoxycholate, and 0.1% SDS. This lysis buffer was supplemented with proteinase and phosphatase inhibitor cocktails (complete Mini Tablets and PhosSTOP, obtained from Roche, Basel, Switzerland). The Pierce BCA Protein Assay Kit (23227, Thermo Fisher Scientific) was used to measure the protein content. Following the manufacturer’s guidelines, 25 µg of total proteins were loaded onto a 4-12% precast polyacrylamide gel (NuPAGE^TM^ 4 bis 12%, Bis-Tris, 1.0–1.5 mm, Mini-Protein-Gel, Invitrogen, NP0321PK2, Waltham, MA, USA) and subsequently transferred onto PVDF 0.45 µm membranes. Proteins were incubated with antibodies provided in Table S3. β-actin served as both the internal loading control and the means of protein quantification. Assays were performed in a minimum of two independent experiments.

###  Reactive oxygen species (ROS) assay

 To determine the amount of ROS produced by GAS5 knockdown, either alone or in conjunction with rituximab treatment, the OxiSelect Intracellular ROS Assay Kit (STA-342, Cell Biolabs, San Diego, CA, USA) was used following the manufacturer’s instructions. As previously mentioned, GAS5 DNAzyme and scramble were transfected into Raji cells either with or without rituximab treatment. Subsequently, the cells were exposed to 2’,7’-Dichlorodihydrofluorescin diacetate (DCFH-DA) during the last hour of the treatment. Following incubation, the cells were rinsed with PBS to eliminate any excess DCFH-DA. Fluorescence measurements were performed with a fluorescent plate reader (Citation 3 image Reader, BioTek) at an excitation wavelength of 485 nm and an emission wavelength of 530 nm. ROS levels were quantified as the relative fluorescence intensity of DCFH-DA and expressed as a fold change in comparison to the control.

###  Caspase-3 activity assay 

 To evaluate caspase-3 enzymatic function following GAS5 knockdown with or without rituximab treatment, cells were treated with a luminogenic substrate specific for caspase-3, containing the DEVD recognition motif (Caspase-Glo® 3/7 Assay Systems, G8091, Promega, USA) for 45 minutes at 37 °C. Luminescence levels are positively associated with caspase activity. Luminescence was quantified using a plate luminometer (Citation 3 image Reader, BioTek), and this assay was conducted in duplicate.

###  Statistical analysis 

 Statistical analysis was performed using GraphPad Prism 8.4.3 (GraphPad Software, Inc., San Diego, CA, USA). Student’s unpaired t-test was used to analyze the statistical significance between the two groups. Analysis of variance (ANOVA) or the equivalent nonparametric test, such as Kruskal-Walis, utilized to evaluate statistically significant differences across more than two experimental conditions. Normality and lognormality tests were conducted before analysis. *P *values < 0.05 were considered significant.

## Results

###  GAS5 interacts with CD20 regulatory proteins 

 Recent studies have demonstrated that GAS5 interacts with various targets, revealing its crucial role in different biological processes and disease pathogenesis.^19^ Kawabata et al demonstrated that activated SMAD2/3 binds directly to the promoter region at the transcription start sites of MS4A1 using chromatin immunoprecipitation (ChIP) assay.^20^ We discovered that GAS5 interacts with SMAD3 and the 5’ untranslated region (UTR) of SMAD2 with a minimum local energy of -42.63 kcal/mol, as determined by the LncRNA2Target and LncRRIsearch databases (Tables S4 and S5).^11,16^ Furthermore, according to the LncRNA2Target and Biogrid databases, GAS5 interacts with CD20 activators such as CDKN2B, STAT3, and EP300 (Figure S1).^11,13^ These transcription factors (TFs) contribute to the modulation of CD20 in the hTF target and mayyanlab databases (Harmonizome 3.0) (Table S5).^13,14^ The obtained data suggest that GAS5 may be critical in regulating CD20 expression levels.

###  GAS5 knockdown increased CD20 expression in Raji cells

**Figure 1:**
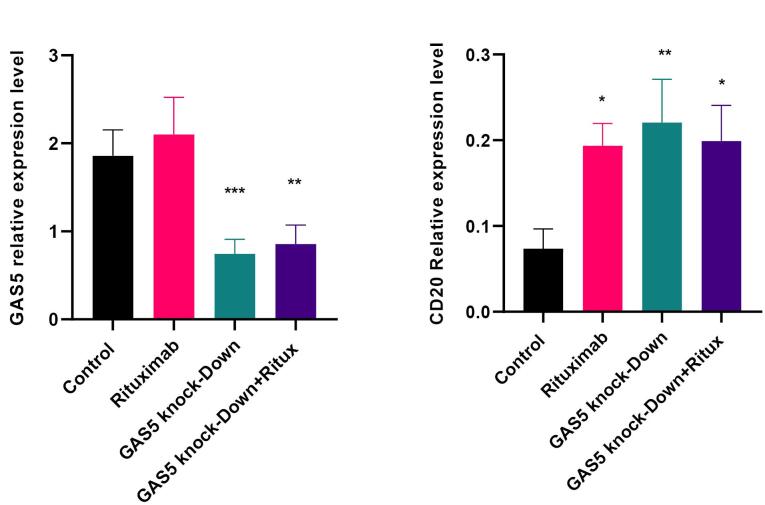
Rituximab treatment and GAS5 knockdown altered the relative expressions of CD20 and GAS5. (a) Raji cells were treated with rituximab at 10 µg /mL for 24 hours and transfected with GAS5 DNAzyme alone and under both conditions. GAS5 expression decreased after transfection with GAS5 DNAzyme alone or after treatment with rituximab compared to the control transfected with scrambled DNAzyme. (b) Knockdown of GAS5 alone and in combination with rituximab increased CD20 expression at the mRNA level. Expression levels were normalized to β-actin as an internal control gene. (***P < 0.001, ** P < 0.01, *P < 0.05)

**Figure 2:**
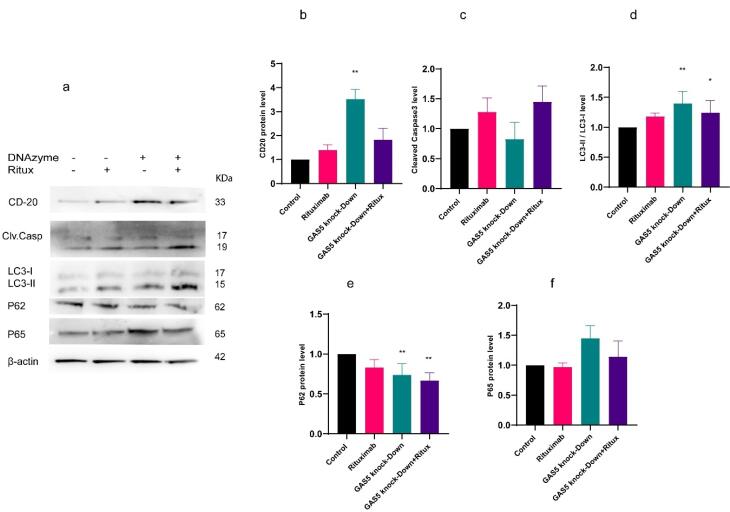
(a) Western blot images indicating the effect of GAS5 knockdown and rituximab treatment on CD20, Cleaved caspase, LC3, P62, and P65 protein levels. (b) At the protein level, GAS5 knockdown significantly increased CD20. (c) Western blot analysis of cleaved caspase 3 indicated the same trend as caspase activity, but not significantly. (d and e) GAS5 knockdown alone or combined with rituximab increased autophagy markers significantly. The levels of LC3II, LC3I, and p62 were evaluated using Western blot analysis. The ratio of LC3 II to LC3I increased following GAS5 knockdown and in combination with rituximab. GAS5 knockdown with or without treatment decreased P62 level, suggesting that GAS5 knockdown may enhance autophagy. (f) Western blot analysis of the p65 protein level showed a similar increasing trend to mRNA after GAS5 knockdown, although the difference was insignificant. All intensity values in the densitometry plots were normalized to the corresponding β-actin value and control. (*P < 0.05, ** P < 0.01, *** P < 0.001)

 To explore the potential regulatory role of GAS5 on CD20 expression, two specific DNAzymes were designed to reduce GAS5 expression. The results indicated that the GAS5-specific DNAzymes reduced their target RNA expression level by over 50%, either alone or in combination with rituximab treatment at 10µg /mL (Figure 1a). The concentration of 10 µg /mL rituximab was utilized based on MTT assay results and prior research.^21^ (Figure S2). The decrease in GAS5 expression resulted in a marked elevation in the CD20 mRNA and protein levels compared to the control. Furthermore, when both rituximab treatment and GAS5 knockdown were applied, the CD20 expression level significantly increased at the mRNA level (Figure 1b, and Figure 2b). These results suggest that GAS5 may negatively regulate CD20 expression in Raji cells.

###  GAS5 knockdown decreased SMAD2 and increased STAT3 expression in Raji cells

**Figure 3:**
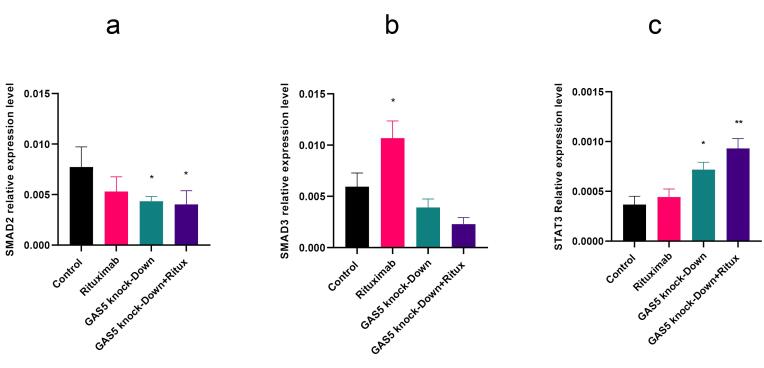
GAS5 knockdown led to a decrease in SMAD2 and SMAD3 levels, while increasing the expression of STAT3. (a) Knockdown of GAS5 with or without rituximab reduced SMAD2 expression statistically significantly. (b) Rituximab treatment significantly increased SMAD3 expression, while GAS5 knockdown led to a decrease in SMAD3 expression that was not statistically significant. (c) GAS5 knockdown alone and with rituximab treatment increased STAT3 expression level. Expression levels were normalized to β-actin as an internal control gene. (**P < 0.01, * P < 0.05)

 Previous research demonstrated that SMAD proteins act as negative regulators of CD20.^22^ Thus, we investigated SMAD expression levels after treatment with rituximab and knockdown of GAS5. The qPCR results indicate a significant decrease in SMAD2 expression following GAS5 downregulation and in the combination treatment. However, GAS5 knockdown did not change SMAD3 expression level (Figure 3a and 3b). Since STAT3 is one of the CD20 TFs, we assessed the expression level of STAT3 after GAS5 downregulation. Results indicated that the knockdown of GAS5 increased STAT3 in both conditions (Figure 3c).

###  GAS5 knockdown decreased the generation of ROS in Raji cells

**Figure 4:**
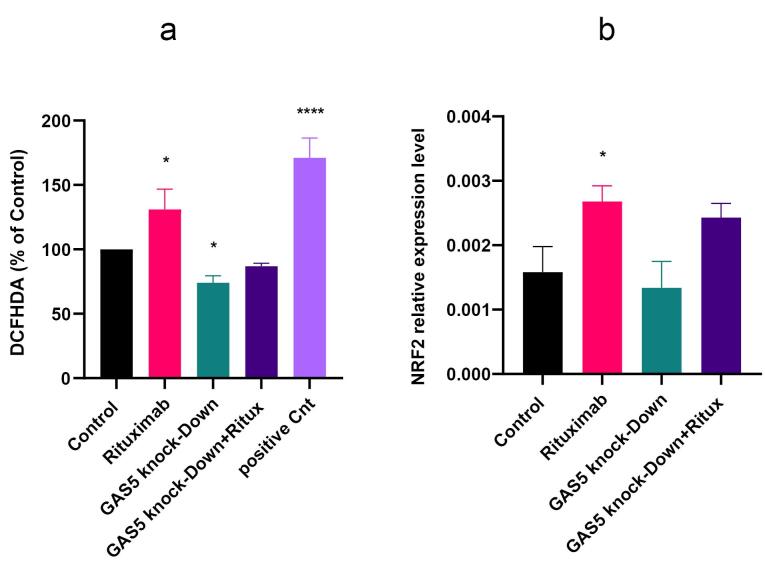
Rituximab and GAS5 knockdown altered the generation of reactive oxygen species (ROS) but did not significantly affect the expression of NRF2. (a) Treatment with rituximab led to a significant increase in ROS production. Conversely, GAS5 knockdown resulted in decreased ROS levels compared to the control. (b) NRF2 expression increased following rituximab treatment but was not altered after GAS5 knockdown. Expression levels were normalized to β-actin as an internal control gene. (****P < 0.0001, * P < 0.05)

 Evidence suggests ROS is linked to various human tumors, such as lymphomas. ROS can shape various cellular functions, including proliferation, differentiation, migration, and apoptosis.^23^ We assessed the impact of GAS5 knockdown and rituximab treatment on the amount of ROS production. Results showed that rituximab increased ROS levels compared to control. However, GAS5 knockdown decreased ROS generation (Figure 4a). This provides evidence that the knockdown of GAS5 protects against ROS formation. Accordingly, we examined the expression level of NRF2 as a TF responsible for the maintenance of cellular redox homeostasis.^24^ Results indicated that NRF2 expression level increased following rituximab treatment (Figure 4b).

###  GAS5 knockdown decreased caspase activity and apoptosis in Raji cells

**Figure 5:**
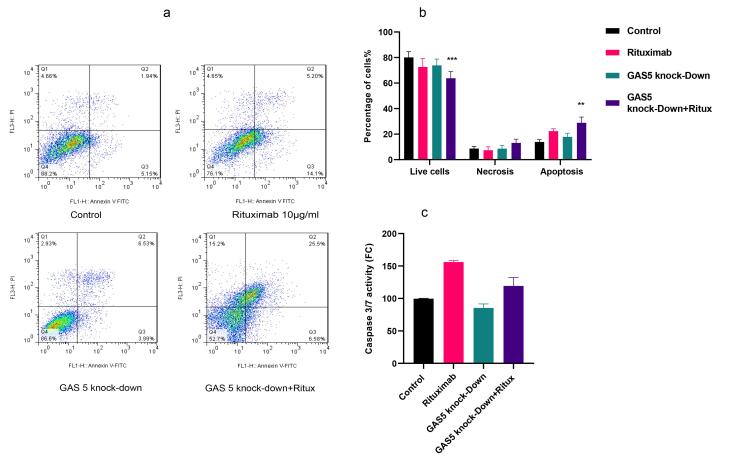
GAS5 Knockdown reduced apoptosis, while the combination treatment increased apoptosis in Raji cells. (a and b) The results of flow cytometry analysis using double staining with Annexin V–FITC and PI revealed that only the combination of rituximab and GAS5 knockdown significantly increased apoptosis. (c) Results indicated that rituximab increased caspase activity either alone or combined with GAS5 knockdown; however, knockdown of GAS5 decreased caspase activity. (***P < 0.001, ** P < 0.01, * P < 0.05)

 Dysregulated cell death, such as apoptosis, is one of the major contributors to cancer pathogenesis. Caspase-3/7 activation is a crucial molecular marker for apoptotic cell death.^25^ Therefore, in this study, we assessed the effect of GAS5 knockdown and rituximab treatment on apoptosis by measuring caspase 3/7 activity and flow cytometry analysis using double staining with Annexin V–FITC. Results showed that in flow cytometry analysis, only knockdown of GAS5 combined with rituximab increased the apoptosis rate significantly (Figure 5a and 5b). However, knockdown of GAS5 decreased caspase activity, while treatment with rituximab alone and in combination with GAS5 knockdown increased caspase activity (Figure 5c). Additionally, western blot analysis showed almost the same trend, although not significantly (Figure 2c). Overall, the data suggest that while knockdown of GAS5 decreased apoptosis, combining the two treatments increased it compared to the control.

###  GAS5 knockdown induced markers of autophagy in Raji cells

 Autophagy is a crucial pathway for maintaining cellular homeostasis and regulating immune responses. It also reduces the production of ROS that activate inflammasomes.^26^ To determine the effect of GAS5 knockdown and rituximab treatment on autophagy in Raji cells, western blot analysis was used to measure LC3-I and LC3-II, as well as protein SQSTM1(p62). LC3 protein has two forms: LC3-I and LC3-II. The process of autophagosome formation and its progress requires the conversion from LC3-I to LC3-II. Moreover, it has been shown that there is a negative correlation between p62 and autophagy activation.^27^ GAS5 knockdown increased the ratio of LC3-II/LC3-I alone and also in combination with rituximab (Figure 2d), but decreased p62 in both conditions (Figure 2e). Our findings indicated that the knockdown of the GAS5 gene, both alone and in combination with rituximab, resulted in the induction of autophagy markers and might improve autophagy in Raji cells.

###  Knockdown of GAS5 induced NF- κB (p65) in Raji cells 

**Figure 6:**
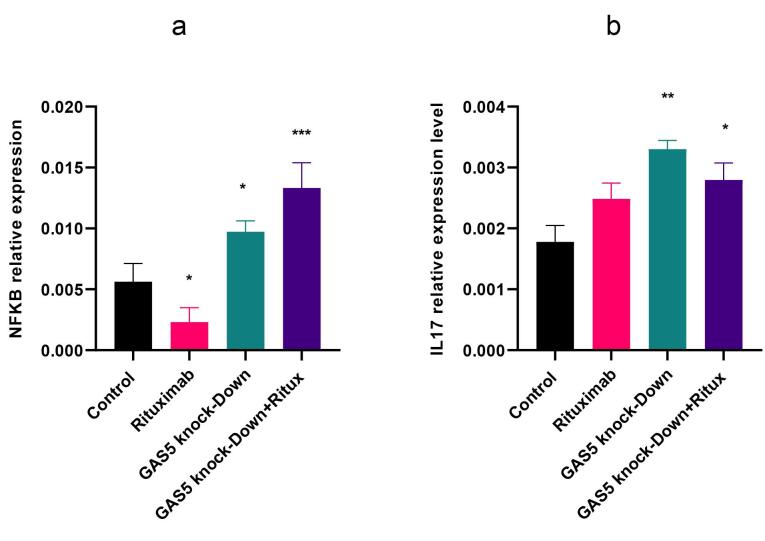
GAS5 knockdown alone or in combination with rituximab significantly increased NF-κB and IL-17 levels. (a) At the mRNA level, rituximab decreased NF-κB expression, but GAS5 knockdown increased it both alone and in combination with rituximab. (b) IL17 expression level increased by GAS5 knockdown alone and in combination with rituximab. Expression levels were normalized to β-actin as an internal control gene. (*P < 0.05, ** P < 0.01, *** P < 0.001)

 NF-κB (Nuclear factor kappa-light-chain-enhancer of activated B cells) is a TF that is essential for the regulation of multiple physiological functions, notably immune responses and inflammatory pathways, cell proliferation, and apoptosis.^28^ In this study, we investigated the effect of GAS5 knockdown and rituximab treatment on NF-κB expression and the protein level of p65, one of the NF-κB subunits. Results indicated that GAS5 downregulation enhanced NF-κB transcript level. However, following rituximab treatment, the expression of NF-κB decreased, but the p65 protein level did not change significantly (Figure 6a and 2f). In addition, GAS5 knockdown alone and combined with rituximab increased interleukin 17 (IL-17) expression (Figure 6b) as an inflammatory cytokine and target gene of NF-κB. Overall, the downregulation of GAS5 increased NF-κB and IL-17. GAS5 may be involved in regulating NF-κB and IL-17 expression.

## Discussion

 Rituximab is the first approved monoclonal antibody against CD20 for treating NHL. NHL is one of the most common forms of lymphoma with a global incidence rate of 5.8 and a mortality rate of 2.6 per 100000 individuals. CD20, encoded by the MS4A1 gene, is a cell surface glycoprotein expressed on B cells.^29,30^ It has been shown that lncRNAs can influence gene expression through different mechanisms, including direct interaction with proteins or indirectly by acting as ceRNAs for miRNAs. GAS5 has been the focus of recent research attention. GAS5 participates in various signaling pathways, including proliferation, apoptosis, autophagy, oxidative stress, and immune cell function in vitro and in vivo, and plays important roles in gene expression regulation.^19,31^

 Our in-silico analysis revealed that GAS5 could interact with SMAD3, the 5’ untranslated region of SMAD2, and CD20 TFs such as STAT3. Kawabata et al found that SMAD2/3 binds directly to the transcription start site of CD20 and represses its expression.^20^ Here, we indicated that GAS5 knockdown decreased SMAD2 and increased STAT3 expression level.

 Similar to other targeted therapeutic approaches, tumor cells may escape treatment by altering the expression or activity of the intended molecular target, such as CD20.^32^ The response rate and resistance to rituximab treatment may be influenced by the level of CD20 protein expressed on the surface of the cells. In this study, for the first time, we showed that GAS5 regulates the expression of CD20. Also, rituximab treatment and knockdown of GAS5 decreased the expression of SMAD2. These results support previous findings that SMAD2 negatively regulates CD20.^22^ Moreover, STAT3, one of the CD20 TFs, increased after GAS5 downregulation, leading to CD20 increased expression. These findings suggest that GAS5 may regulate CD20 expression and could be targeted for therapeutic intervention in diseases involving CD20 dysregulation. We must recognize the constraints of this study, which limited our evaluation to the possible direct induction of apoptosis by rituximab. Further research utilizing RNA-protein precipitation is necessary to clarify the mechanism by which GAS5 interacts with CD20 regulatory proteins. Additionally, the dosage of rituximab and the stage of the disease may affect these interactions.

 GAS5 may play a role in drug response by modulating the expression of CD20 or through various other mechanisms involved in this process. The reaction of cells following treatment is determined by a range of cellular processes that assess cell survival, including apoptosis, autophagy, inflammation, and redox state.^33^ Thus, the impact of GAS5 knockdown on apoptosis, ROS production, and autophagy was investigated.

 The regulation of ROS levels is crucial for controlling apoptosis, and various factors can influence ROS generation.^34^ Our results showed that rituximab treatment increased ROS levels, consistent with previous studies, demonstrating that anti-CD20 antibodies can stimulate ROS release through NOX2 mediation in monocytes.^35^ Interestingly, we also observed that the reduction in GAS5 expression led to a decrease in ROS generation. We hypothesize that GAS5 may act as a regulator of ROS generation. Also, the results indicated that treatment with rituximab increased NRF2 expression by inducing higher levels of ROS. As previous studies have shown, ROS activates NRF2, and in turn, NRF2 can activate its gene expression in an autoregulatory mechanism.^36^ Similar to our results, studies demonstrated that GAS5 knockdown alleviates oxidative stress in ox-LDL-treated THP-1 cells,^37^ and mitochondrial ROS in PC-12 cells, but increases NRF2 expression.^38^ However, in contrast to our results, Chen et al’s research showed that silencing GAS5 in A375 cells resulted in the production of ROS.^39^ These findings, as well as our results, suggest that the role of GAS5 in regulating ROS levels and oxidative stress may be complex and context-dependent. Additional investigations are needed to gain a comprehensive understanding of the molecular processes and clinical implications of targeting GAS5 in regulating oxidative stress.

 The present study demonstrated that rituximab increased caspase activity, however, GAS5 knockdown decreased it. Similar to our findings, a study investigating the effects of rituximab treatment on B-NHL cells, indicated that rituximab treatment triggered several apoptotic events, including internucleosomal DNA fragmentation and caspase–3–like activity.^40^ In addition, in vivo experiments conducted in patients with chronic lymphocytic leukemia (CLL) have shown the activation of caspase9/3.^41^ Moreover, the findings of this study align with earlier reports that have shown the involvement of GAS5 in the regulation of caspase activity. Separate studies conducted by Tang et al in 2019 and Cao et al in 2021 showed that silencing GAS5 can suppress the cleavage of caspase 3 in vascular smooth muscle cells and hypoxia-treated cells, respectively.^42,43^ These findings suggest that GAS5 may play a role in regulating caspase activity and cell survival in various contexts. Combining GAS5 knockdown with rituximab treatment increased apoptosis; however, it resulted in a reduction of caspase activity compared to rituximab treatment alone. It is important to note that we only evaluated the direct induction of apoptosis by rituximab. The apoptosis process may be influenced by CD20 expression, as higher rituximab binding may occur with increased CD20 levels following GAS5 downregulation. Additionally, the timing of apoptosis testing warrants further investigation in future studies.

 Autophagy has been shown to participate in physiological processes ranging from cell differentiation and development, tumor suppression, innate and adaptive immunity, to cell death. ^44^ Rituximab and its conjugates can activate autophagy by inhibiting the mTOR pathway, as demonstrated in some studies.^45,46^ Our results showed that GAS5 knockdown may enhance autophagy. However, the role of GAS5 in autophagy regulation remains controversial, with previous studies demonstrating conflicting outcomes. For instance, Liang et al have corroborated our findings by reporting that the inhibition of GAS5 resulted in the suppression of cell apoptosis and the initiation of autophagy flux in ox-LDL-treated human aortic endothelial cells.^47^ Furthermore, another study indicated that the downregulation of GAS5 stimulates autophagy in a model of atherosclerosis.^48^ In contrast, Li et al have demonstrated that GAS5 downregulation inhibits autophagy by regulating miR-23a in an ATG3-dependent manner.^49^ In glioma and NSCLC models, GAS5 appears to suppress excessive autophagy and enhance cisplatin sensitivity by activating mTOR signaling.^50,51^ This apparent contradiction may stem from context-dependent regulatory mechanisms and reflect cell-type specificity, disease context, or interactions with distinct miRNA axes, which modulate autophagy-related proteins.^51,52^ Thus, GAS5 has been implicated in both promoting and inhibiting autophagy through various molecular pathways. Apoptosis and autophagy are two distinct types of programmed cell death that can occur simultaneously or separately. Their interaction might be cooperative or oppositional, depending on the cellular conditions.^53^ In rituximab treatment, the association between these two processes is complex and not completely known, but it may significantly alter the drug’s cytotoxic effects. Wang et al in 2018, showed that autophagy inhibition using chloroquine reduces the number of apoptotic cells induced by rituximab conjugated drug in B-cell lymphoma cell lines.^45^ While the findings of this study suggest that rituximab treatment combined with GAS5 knockdown may enhance autophagy and apoptosis compared to control, further research is needed to elucidate the role of GAS5 in autophagy regulation and its potential implications in lymphoma.

 Inflammation plays an important role in tumorigenesis and metastasis through various mechanisms, and NF-κB is one of the major drivers of inflammation.^54^ Also, it is important to note that, the transcription of NF-κB-dependent genes affects the levels of ROS in the cell, and in turn, NF-κB activity is also controlled by ROS levels. Depending on the context, ROS can both activate and inhibit NF-κB signaling.^55^ Here, we observed that GAS5 can impact the inflammatory state of the cells by modulating NF-κB and IL-17 as the target gene of NF-κB.^56^ Consistent with our results, studies conducted by Su et al and Baumschabl et al revealed a negative relationship between GAS5 and NF-κB expression.^57,58^ Likewise, in a mouse model of rheumatoid arthritis, GAS5 siRNA enhanced NF-κB compared to the control group.^59^ According to our findings and previous studies, GAS5 seems important in regulating inflammatory pathways. However, the knockdown of GAS5 combined with rituximab increased the expression levels of NF-κB and IL-17, but did not significantly affect the p65 protein level, although it showed the same increasing trend. To understand this discrepancy, it is important to note that p65 is one of the NF-κB subunits; however, measuring its expression reflects the overall NF-κB level. Additionally, IL-17 expression may be influenced by factors beyond NF-κB activation alone; therefore, further investigation is needed. The relationship between ROS, apoptosis, inflammation, and autophagy is complex and dependent on the studied microenvironment.^60^ In this study, our results demonstrated that GAS5 knockdown reduced apoptosis and ROS, but increased autophagy and inflammation. However, GAS5 knockdown combined with rituximab resulted in increased levels of apoptosis, autophagy, and inflammation, along with a decreasing trend in ROS levels. ROS has complex interactions with different pathways. Its level determines its role in apoptosis, inflammation, and autophagy. Low levels of ROS can activate cell survival signaling pathways, while high levels can activate cell death signaling pathways.^61^ The reduced level of apoptosis observed in the combination treatment, as opposed to rituximab treatment alone, can be associated not only with the downregulation of GAS5 as a tumor suppressor but also with lower levels of ROS. In addition, ROS plays a role in both the initiation and resolution of inflammation, plus the induction and inhibition of autophagy.^62,63^ Alternatively, it is important to recognize that autophagy plays a crucial role in maintaining redox homeostasis. Numerous anti-cancer agents trigger autophagy through ROS, a process that may result in either the emergence of drug resistance or the activation of autophagic and/or apoptotic pathways. To achieve cytotoxicity, cancer therapy strategies are now attempting to take advantage of ROS and autophagy.^62,64^

 Our results paint a complex picture where GAS5 knockdown influences ROS, apoptosis, autophagy, and inflammation. Interpreting these results requires an understanding of the crosstalk between these processes. The observed upregulation of autophagy could be a double-edged sword.^65^ Initially, it likely serves a cytoprotective function, mitigating damage by clearing dysfunctional components and reducing ROS levels, which could account for the decrease in apoptosis following GAS5 knockdown alone. However, the introduction of rituximab alters this dynamic. The combination therapy imposes a heightened state of cellular stress, under which the enhanced autophagic response may become excessive. Excessive autophagy itself may start contributing to cell death, helping to drive the increased apoptosis observed. This shift from pro-survival to pro-death function is a crucial concept in cancer biology.^66^ Several other studies have shown that a well-established molecular crosstalk connects autophagy and apoptosis. For instance, caspase-mediated cleavage of key proteins like Beclin-1 and their interaction with BCL-2 regulates initiation or inhibition of autophagy. This balance is critically relevant in lymphomagenesis.^67^ The observed decreasing trend in ROS under combination treatment could be the consequence of upregulated autophagy actively recycling oxidized components to maintain redox homeostasis, which in turn can influence signaling pathways that decide the cell’s fate.^66^ NF-κB and ROS operate in a synergistic relationship, each potentiating the activity of the other. NF-κB is both a source and a target of ROS and can transcriptionally regulate antioxidative and pro-oxidant genes as well as pro-survival autophagy genes.^68^ Normally, autophagy can dampen inflammatory signaling by degrading inflammasome components. Our result showing increased NF-κB/IL-17 expression alongside increased autophagy in the combination treatment group presents a paradox that may be unique to the lymphoma microenvironment. It suggests that GAS5 knockdown might create a hyper-inflammatory, stress-induced state where autophagy is unable to resolve the inflammation it may be helping to fuel.

 Overall, to evaluate the clinical benefits of GAS5 in enhancing the cytotoxic effects of rituximab, further research is necessary. This research should thoroughly investigate how GAS5 regulates both direct and indirect apoptosis induction such as CDC and ADCC by rituximab. Furthermore, it should investigate the roles of autophagy, ROS, and inflammatory pathways in both rituximab-resistant and sensitive lymphoma cell lines, as well as in patient samples from various tumor stages.

## Conclusion

 The findings of the current study suggest that GAS5 may have an indirect regulatory role in the expression of CD20 in B cells, through the modulation of TFs such as SMAD2 and STAT3. To the best of our knowledge, this is the first work demonstrating the possible regulatory role of GAS5 on CD20 expression in Raji cells, besides its effect on autophagy, apoptosis, and inflammation that can influence the cytotoxicity rate of rituximab in Raji cells. The intricate relationship between these cellular pathways underscores the complexity of GAS5’s influence on treatment response and tumor behavior. Thus, GAS5 may be an interesting therapeutic target in lymphoma or other B-cell-related diseases to enhance the efficiency of chemotherapeutic agents like rituximab.

## Competing Interests

 The authors declare that there are no conflicts of interest related to this study.

## Data Availability Statement

 M. Behmanesh and M. Rojhannezhad had full access to all data in the study and take responsibility for the integrity of the data and accuracy of the data analysis.

## Ethical Approval

 This study was conducted in accordance with ethical standards and did not include human subjects or biological specimens. Likewise, no live animals were involved in any experimental procedures. All of the experiments conducted were approved by the Tarbiat Modares Research Ethics Committee (IR.MODARES.RES.1398.078).

## Supplementary Files


Supplementary file contains Table S1-S5 and Figure S1 and S2.
