# Supplementary material for: GAS5 Long Noncoding RNA Regulates CD20 Expression and Rituximab Response
Source: Adv Pharm Bull. 2025 Oct 11;15(4):928–38. doi: 10.34172/apb.025.45822 (PMC12980193; doi:10.34172/apb.025.45822)
Supplement: Supplementary file 1 — Supplementary file contains Table S1-S5 and Figure S1 and S2. [file apb-15-928-s001.pdf]

Adv Pharm Bull. 2025;15(4):928-S1
doi: 10.34172/apb.025.45822
https://apb.tbzmed.ac.ir

Supplementary file 1

Table S1. Sequence of DNAzymes used against GAS5.

DNAzyme	Sequence	Minimum free energy	MFE plain structure drawing
GAS5	CTTGCTCCACAA-GGCTAGCTACAACGA - AGTGTAGTCA	-7.10 kcal/mol	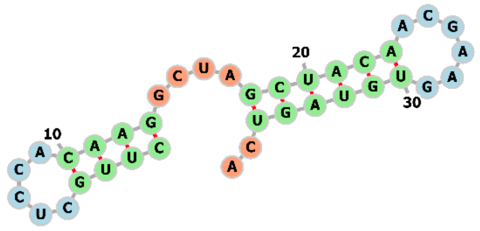
GAS5	GTGAGGCAAGAA-GGCTAGCTACAACGA - CCTTTCAAGC	-3.10 kcal/mol	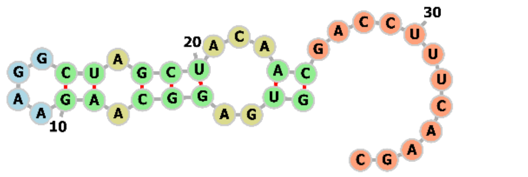
Scramble	TCTATAAGCA-GGCTTGCTATAACGA - GTCAAGCTG	-8.30 kcal/mol	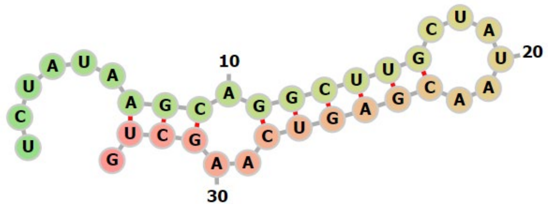

Table S2. Sequence of primers against the human genes analyzed by qPCR.

**Gene Symbol**	**Sequences**	**Amplification length**
β-actin	F:TAAGGAGAAGCTGTGCTACGTC	**261bp**
	R:AGCACTGTGTTGGCGTACAGGTC	
Growth arrest specific 5 (GAS5)	F: CACAACAAGCAAGCATGCAG	**169 bp**
	R:TCTTCTTGTGCCATGAGACTCC	
Membrane spanning 4-domains A1 (MS4A1) or CD20	F:TGCTCCAGACCCAAATCTAAC	**296 bp**
	R: CTCTTGGAAGCTATGAACAC	
SMAD Family Member2 (SMAD2)	F:GTCAAAAGTACGATGTGGTTCC	**266 bp**
	R:CACAGGTAAGAGCAAACAAGG	
Nuclear factor kappa B subunit 1(NF-κB)	F:TACTCTGGCGCAGAAATTAGGTC	**265bp**
	R:ACTGTCTCGGAGCTCGTCTATTTG	
Nuclear factor, erythroid 2 like 2 (NRF2)	F: CATTGAGCAAGTTTGGGAGGAG	**233bp**
	R: CTGTGGAGAGGATGCTGCTGAAGG	
Interleukin 17A (IL17A)	F: CTTCCCCCGGACTGTGATGGTCAA	**212bp**
	R: TCATGTGGTAGTCCACGTTCCCAT	

Table S3. List of antibodies for western blot (WB).

Antibody	supplier	Clone / Cat. no.	Concentration
β-actin	SCB	2418	1:4000
Cleaved caspase-3	CST	5A1E / 9664	1:1000
LC3B	CST	D11 / 3868	1:1000
SQSTM1/p62	CST	5114	1:1000
NF-κB (p65)	CST	(C-20): sc-372	1:1000
CD20	PT	60271-1-Ig	1:20000
**Secondary antibody for WB**			
Goat anti -rabbit IgG (HRP)	CST	7074	1:2000

CST: Cell Signaling Technology; PT: Proteintech, SCB: Santa Cruz Biotechnology

Table S4. Prediction of lncRNA-RNA interaction of GAS5 and SMAD2 5'UTR.

Local base-pairing interactions of GAS5 and SMAD2.

Gene name	Number of base pair	Sequence	Energy
GAS5 (ENST00000431268)	From nucleotide 605 to 709	GCCCAG AATGGAGTGCAGCGGCACTATCAGTTCACTGCAACCTCTGCCTCCCAGGTTCAA GGAATTCTCCTGCCTCAGCCTCTTGAGTAGCTGGGATTACAGGCA	-42.63 kcal/mol
SMAD2 (ENST00000262160)	From nucleotide 26576 to 26680 (5'UTR)	TGCCTATGATCCCGCTACTTGGGAGGCTGAGGCAGAAGATTGGCTTGAACCTGA GAAGTGGAGGTTGCAGTGAGCCTAGATGTGCCACTGCACTCCAGCCTGGGC	

Table S5: GAS5 interacts with CD20 regulatory proteins.

Gene	Interactions	Experimental system	Database	Ref
GAS5	SMAD2	5' UTR	LncRRIsearch: http://rtools.cbrc.jp/LncRRIsearch/	Tsukasa Fukunaga et al., 2019.[1]
GAS5	SMAD3	Luciferase reporter assays	LncRNA2Target: http://bio-annotation.cn/lncrna2target/	Cheng L et al., 2021.[2]
GAS5	CDKN2B, STAT3, EP300	Luciferase reporter assays	LncRNA2Target: http://bio-annotation.cn/lncrna2target/ Biogrid: https://thebiogrid.org/	Cheng L et al., 2021.[2] Oughtred R et al., 2020.[3]
CD20	CDKN2B, STAT3, EP300	RNA pull-down assay	hTF target: https://bio.tools/hTFtarget, Harmonizome: https://maayanlab.cloud/Harmonizome/	Zhang Q et al., 2020.[4] Diamant I, et al.,2024.[5]

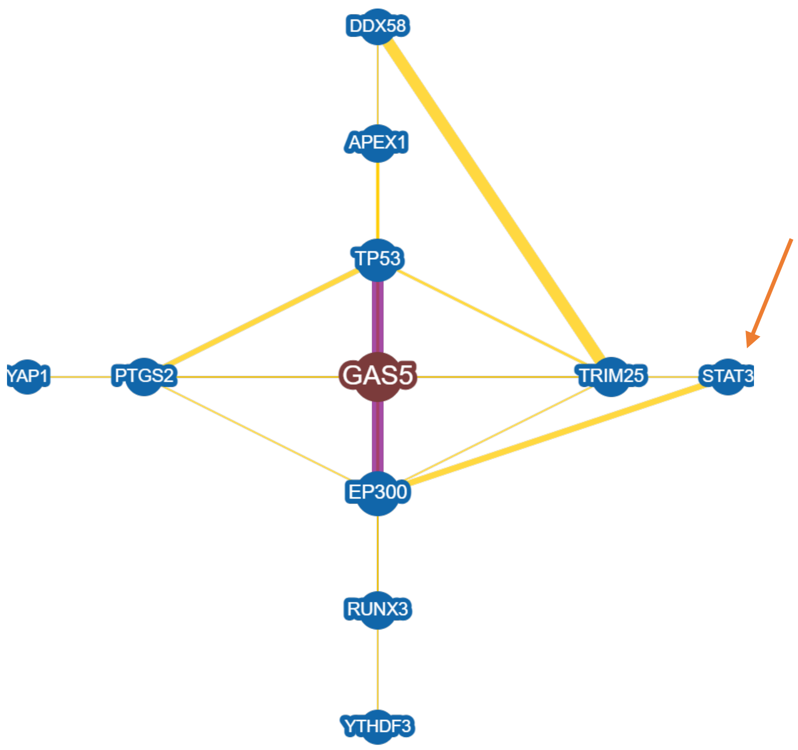

Figure S1. Biogrid network interaction of GAS5 and some of CD20 Transcription factors such as STAT3.

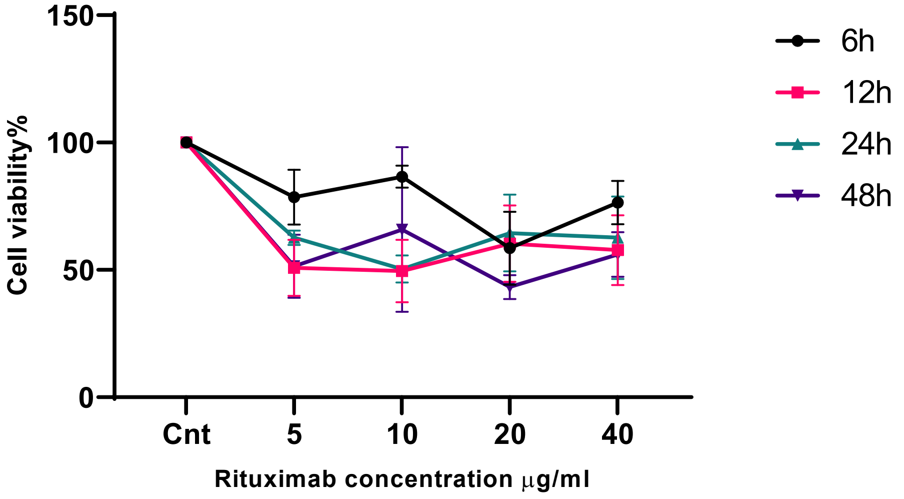

Figure S2. Selection of 10µg/ml rituximab for 24h based on MTT assay.

MTT assay. Raji cells were treated with different concentrations of rituximab at various time points. Rituximab at 10µg/ml for 24 hours reduced cell viability to approximately 50%. This concentration was chosen for further experiments; the data belong to three biological replicates.

References:

1. Fukunaga T, Iwakiri J, Ono Y, Hamada M. LncRRIsearch: a web server for lncRNA-RNA interaction prediction integrated with tissue-specific expression and subcellular localization data. *Frontiers in genetics* 2019;10:462.

2. Cheng L, Wang P, Tian R, Wang S, Guo Q, Luo M, et al. LncRNA2Target v2. 0: a comprehensive database for target genes of lncRNAs in human and mouse. *Nucleic acids research* 2019;47(D1):D140-D4. https://doi.org/10.1093/nar/gky1051

3. Oughtred R, Rust J, Chang C, Breitkreutz BJ, Stark C, Willems A, et al. The BioGRID database: A comprehensive biomedical resource of curated protein, genetic, and chemical interactions. *Protein Science* 2021;30(1):187-200. https://doi.org/10.1002/pro.3978

4. Zhang Q, Liu W, Zhang H-M, Xie G-Y, Miao Y-R, Xia M, Guo A-Y. hTFtarget: a comprehensive database for regulations of human transcription factors and their targets. *Genomics, proteomics & bioinformatics* 2020;18(2):120-8. . https://doi.org/10.1016/j.gpb.2019.09.006

5. Diamant I, Clarke DJ, Evangelista JE, Lingam N, Ma'ayan A. Harmonizome 3.0: integrated knowledge about genes and proteins from diverse multi-omics resources. *Nucleic Acids Research* 2025;53(D1):D1016-D28. https://doi.org/10.1093/nar/gkae1080
